# Supplementary material for: Automated prediction of site and sequence of protein modification with ATRP initiators
Source: PLoS One. 2022 Sep 19;17(9):e0274606. doi: 10.1371/journal.pone.0274606 (PMC9484671; doi:10.1371/journal.pone.0274606)
Supplement: S1 File — (DOCX) [file pone.0274606.s001.docx]

**Installing Anaconda as a pre-requisite in Windows OS.** To run PRELYM, we first installed Anaconda, to create a virtual environment. The 64-Bit Graphical Installer for Anaconda was downloaded from [www.anaconda.com/products/individual#windows](http://www.anaconda.com/products/individual#windows). On-screen instructions were followed and upon successful installation, the Anaconda prompt will be available in the search bar.

**Preparation of input files for PRELYM.** To execute PRELYM, three input files are necessary. As an example, the process of preparing these files will be explained using the protein Avidin. The PDB file for Avidin (PDB ID: 2AVI) was downloaded from the Protein Databank ([www.rcsb.org](http://www.rcsb.org)). Removal of water molecules and the addition of missing atoms was accomplished using Discovery Studio. The modified PDB file was then saved as a new file, named 2AVI_modified.pdb, and was the first input file.

To generate the second input file, we used the previously prepared first input file and MolProbity (<http://molprobity.biochem.duke.edu>). MolProbity is a general-purpose web server that offers quality validation for 3D structures of proteins, nucleic acids, and complexes[1]. For this study, MolProbity allowed us to add and fully optimise bond lengths for all hydrogen atoms present in the protein structure. Once this process was completed, we obtained a new PDB file with the optimised hydrogen atoms named 2AVI_modifiedFH.pdb.

For the third input file, we prepared a .pqr file using the APBS server (<http://server.possionboltzmann.org>), using the PDB2PQR job configuration tool. Here, the first input file was used, and appropriate settings for pH were selected. In our study, we used pH 8.0 to be aligned with the experimental conditions used in previous studies. Prior to initiating the process, we confirmed that the ‘create an APBS input file’ was selected. Upon completion, a list of files was generated. As the third input, the PQR file was downloaded and renamed to 2AVI.pqr.

**Executing PRELYM.** To execute PRELYM, the three input files generated previously along with the two program files (.py and .yml, freely available for download at GitHub <https://github.com/scarmali/PRELYM> ) were placed in a common folder (Figure S1).


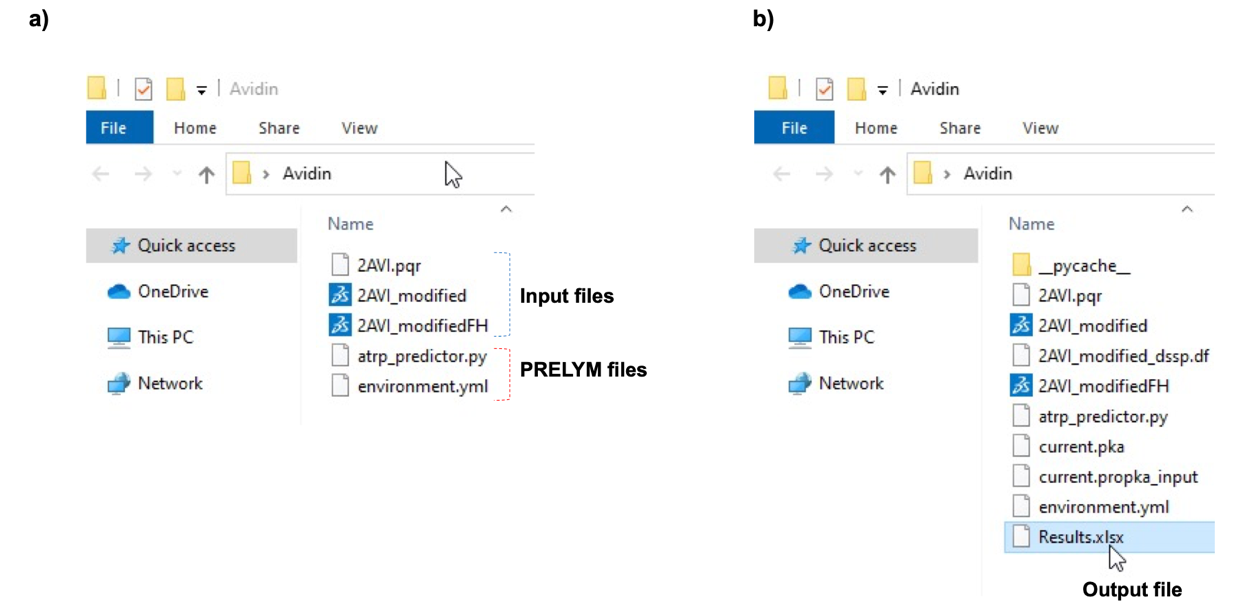


Figure S1 Folder view with PRELYM input and output files. In the common folder a) three generated input files and the two PRELYM program files are placed for PRELYM execution; b) after running PRELYM, a ‘results’ spreadsheet is generated as the output.

In the Anaconda Prompt, the current working directory was set to the path where the common folder with the required files was located. The next step involved creating a virtual environment for the local computer and is only necessary to be done once. In the Anaconda Prompt, *conda env create -f environment.yml* was typed. When prompted, *y* was selected and upon completion, a prompt to either activate or deactivate conda appeared. Conda activation led to a change from base to ATRP environment, indicating the successful creation of the local environment (Figure S2). Python was then used in Anaconda to execute PRELYM. In the Anaconda prompt, PRELYM was called by typing *atrp_predictor import decision_tree* in the command line. The function *decision_tree* was then provided with four arguments: the three input files generated previously, and the selected probe radius (Figure S3). For ATRP initiator modifications, the probe radius was set to 4.2 Å. PRELYM will was then run for a period dependent on the protein size. Once completed, a ‘Results.xlsx’ output file was automatically generated in the common folder (Figure S1).


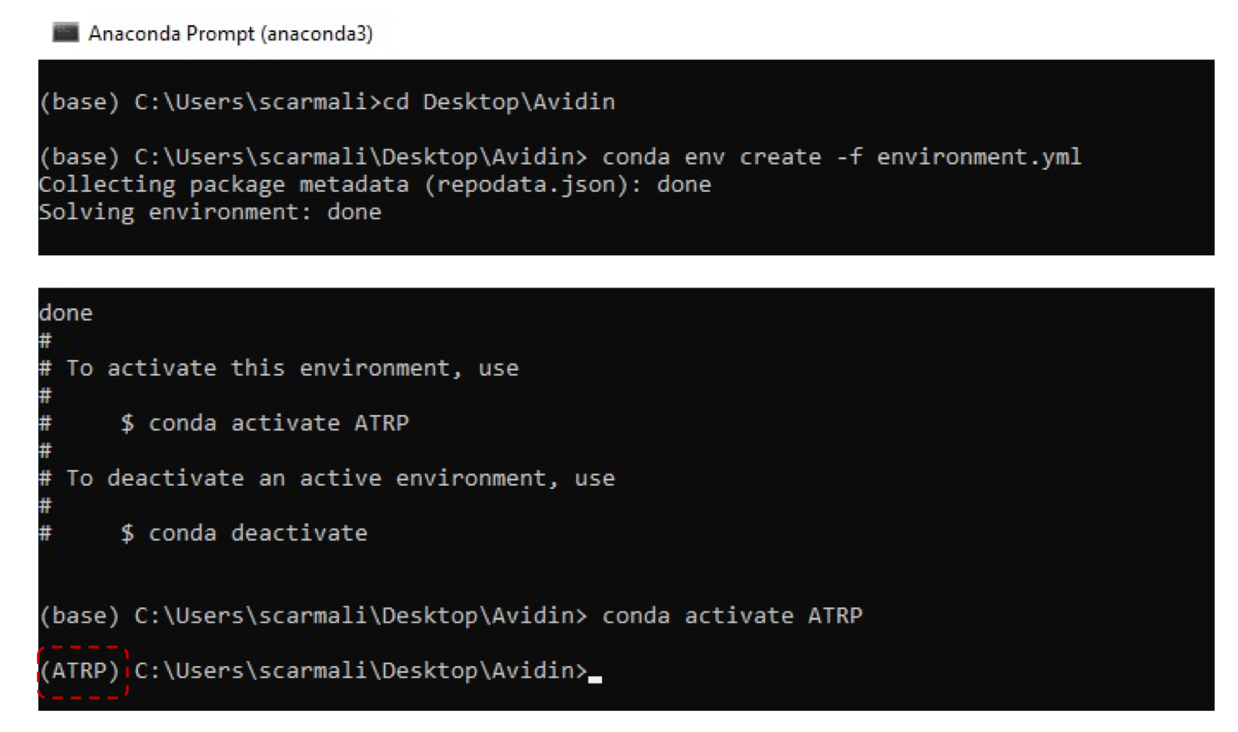


Figure S2 Creation and activation of local environment. Anaconda Prompt showing creation and activation of local environment. Highlighted in red indicates the successful activation of the local environment from base to ATRP.


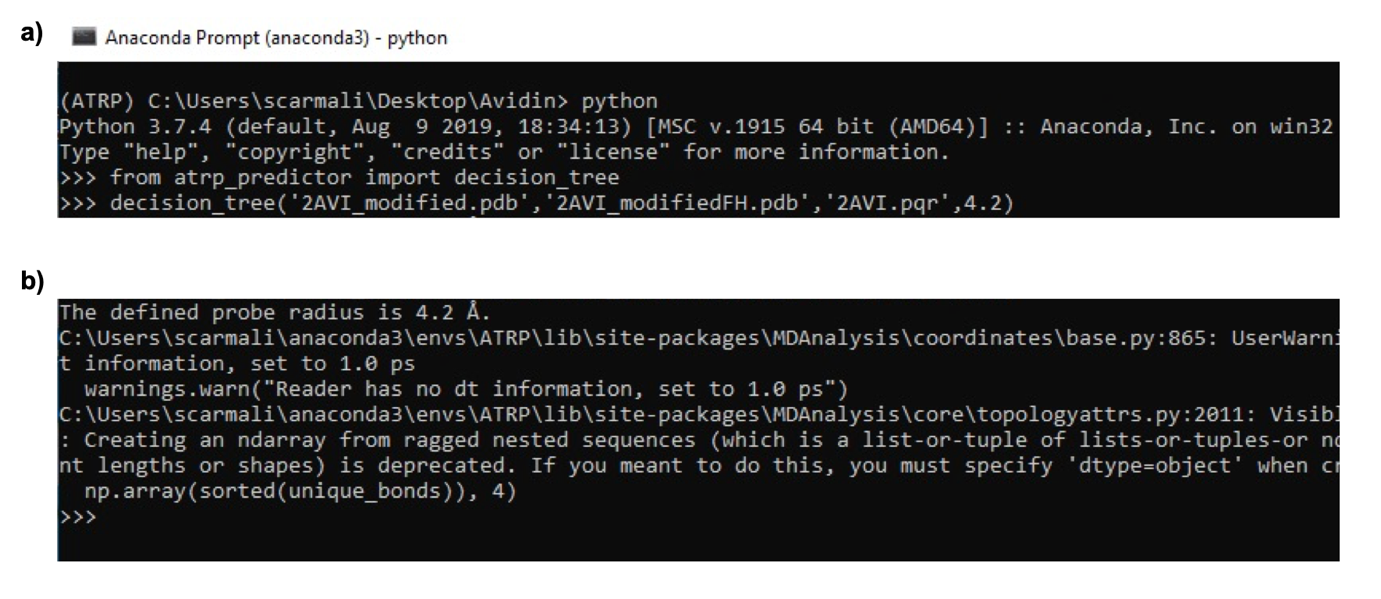


Figure S3 PRELYM execution using Python 3.7.4 in the Anaconda Prompt. In the Anaconda Prompt, PRELYM is executed by a) calling the decision tree function with the required arguments. Upon running, PRELYM will b) confirm the defined probe radius and output a series of user warnings before generating a ‘results’ spreadsheet file in the common folder.

**REFERENCES**

1. Williams CJ, Headd JJ, Moriarty NW, Prisant MG, Videau LL, Deis LN, et al. MolProbity: More and better reference data for improved all-atom structure validation. Protein Science. 2018;27(1):293-315.
