# Supplementary material for: Automated prediction of site and sequence of protein modification with ATRP initiators
Source: PLoS One. 2022 Sep 19;17(9):e0274606. doi: 10.1371/journal.pone.0274606 (PMC9484671; doi:10.1371/journal.pone.0274606)
Supplement: S2 File — Estimation of probe radius for chain transfer agents. (DOCX) [file pone.0274606.s002.docx]

**
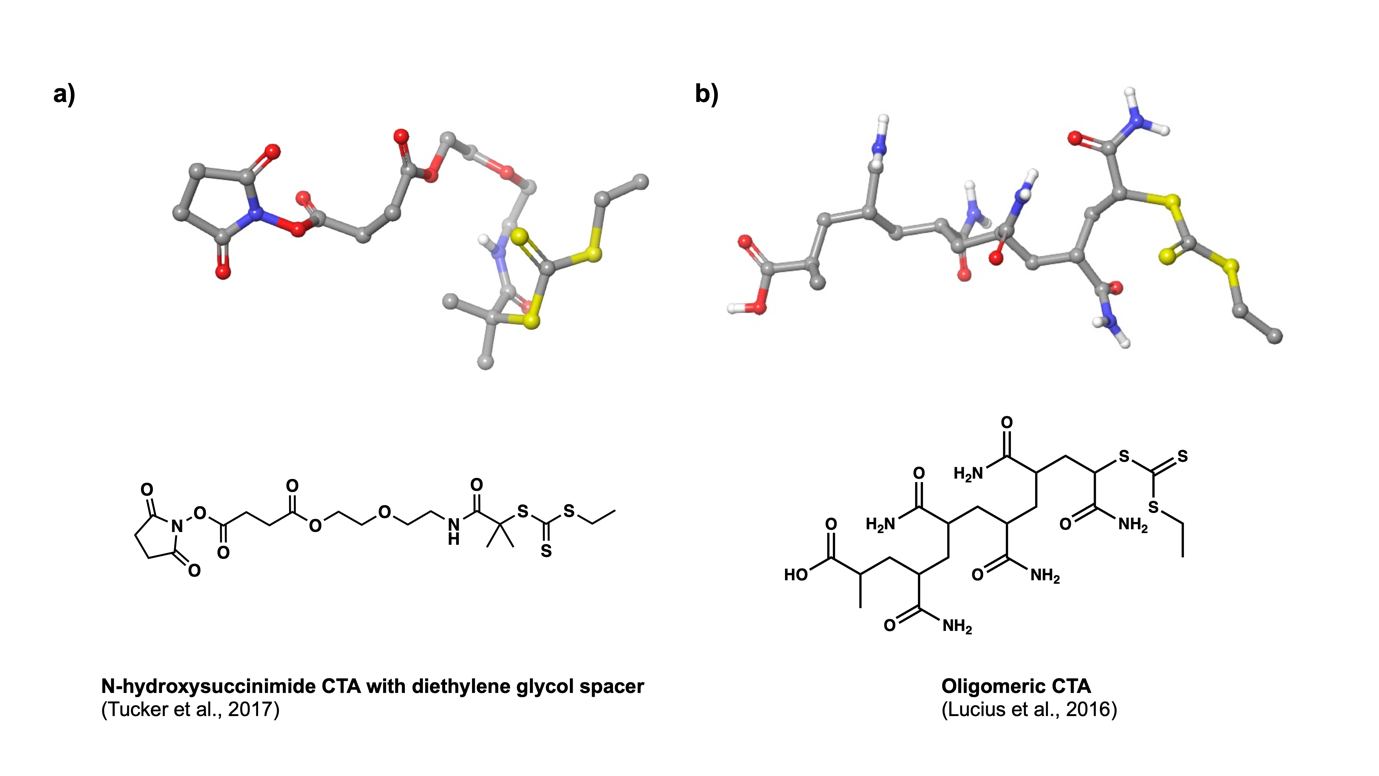
**

Figure S4 Chain transfer agents used for surface-initiated RAFT of lysozyme. 3D and chemical structures of a) *N*-hydroxysuccinimide CTA with diethylene glycol spacer used for photoinduced electron/energy transfer-reversible addition-fragmentation chain transfer (PET-RAFT) polymerisation and b) oligomeric RAFT CTA with 5 acrylamide units.

**Estimation of probe radius for chain transfer agents.** CTA chemical structures were first built using Maestro 2D Sketcher and imported into the main workspace area for 3D transformation.[1] Geometry minimisation was carried out using the default force field (OPLS_2005).[2] End-to-end distances were determined using the “measure” tool in Maestro. The obtained distance was considered as the theoretical diameter of the corresponding CTA, and the value divided by 2 was used as the probe radius for PRELYM calculations.

**REFERENCES**

1. Schrödinger Release 2022-1: Maestro. New York, NY: Schrödinger, LLC; 2021.

2. Shivakumar D, Williams J, Wu Y, Damm W, Shelley J, Sherman W. Prediction of Absolute Solvation Free Energies using Molecular Dynamics Free Energy Perturbation and the OPLS Force Field. Journal of Chemical Theory and Computation. 2010;6(5):1509-19.
