# Supplementary material for: Automated prediction of site and sequence of protein modification with ATRP initiators
Source: PLoS One. 2022 Sep 19;17(9):e0274606. doi: 10.1371/journal.pone.0274606 (PMC9484671; doi:10.1371/journal.pone.0274606)
Supplement: S1 Table — (DOCX) [file pone.0274606.s003.docx]

S1 Table PRELYM results for amine-ATRP initiator interactions on the surface of lysozyme. Shaded in grey are the experimental reactivity data for lysozyme from site modification studies with *N*-hydroxysuccinimide ATRP initiator.[1]

| **Chain** | **Residue** | **-NH2 Group** | **ESA (Å^2^)** | **pKa** | **Secondary Structure** | **H-Donor** | **Area of Lower Charge** | **Reactivity** | |
| --- | --- | --- | --- | --- | --- | --- | --- | --- | --- |
|  |  |  |  |  |  |  |  | **Predicted** | **Experimental** |
| A | K1 | α | 136.34 | 7.43 | Coil | No | Yes | fast-reacting | *not determined* |
|  | K1 | ε | 136.34 | 11.40 | Coil | No | Yes | fast-reacting | *not determined* |
|  | K13 | ε | 69.02 | 11.54 | Helix | Yes | Yes | slow-reacting | slow-reacting |
|  | K33 | ε | 75.91 | 10.14 | Helix | Yes | Yes | slow-reacting | slow-reacting |
|  | K96 | ε | 38.36 | 10.09 | Helix | Yes | Yes | non-reacting | non-reacting |
|  | K97 | ε | 145.03 | 10.45 | Helix | No | Yes | slow-reacting | slow-reacting |
|  | K116 | ε | 134.68 | 10.06 | Coil | Yes | Yes | fast-reacting | fast-reacting |

**REFERENCES**

1. Carmali S, Murata H, Amemiya E, Matyjaszewski K, Russell AJ. Tertiary Structure-Based Prediction of How ATRP Initiators React with Proteins. ACS Biomaterials Science & Engineering. 2017;3(9):2086-97.
