# Supplementary material for: Automated prediction of site and sequence of protein modification with ATRP initiators
Source: PLoS One. 2022 Sep 19;17(9):e0274606. doi: 10.1371/journal.pone.0274606 (PMC9484671; doi:10.1371/journal.pone.0274606)
Supplement: S2 Table — (DOCX) [file pone.0274606.s004.docx]

S2 Table PRELYM results for amine interactions on the surface of lysozyme using a probe radius equivalent to the hydrodynamic radius of *N*-hydroxysuccinimide RAFT CTA (7 Å; see S2 File.). Shaded in grey are experimental data for lysozyme from site modification studies with *N*-hydroxysuccinimide RAFT CTA.[1]

| **Chain** | **Residue** | **-NH2 Group** | **ESA (Å^2^)** | **pKa** | **Secondary Structure** | **H-Donor** | **Area of Lower Charge** | **Reactivity** | |
| --- | --- | --- | --- | --- | --- | --- | --- | --- | --- |
|  |  |  |  |  |  |  |  | **Predicted** | **Experimental** |
| A | K1 | α | 173.27 | 7.43 | Coil | No | Yes | fast-reacting | *not determined* |
|  | K1 | ε | 173.27 | 11.40 | Coil | No | Yes | fast-reacting | *not determined* |
|  | K13 | ε | 70.84 | 11.54 | Helix | Yes | Yes | slow-reacting | *not determined* |
|  | K33 | ε | 95.83 | 10.14 | Helix | Yes | Yes | slow-reacting | modified |
|  | K96 | ε | 35.64 | 10.09 | Helix | Yes | Yes | non-reacting | *not determined* |
|  | K97 | ε | 174.27 | 10.45 | Helix | No | Yes | slow-reacting | modified |
|  | K116 | ε | 185.04 | 10.06 | Coil | Yes | Yes | fast-reacting | *not determined* |

**REFERENCES**

1. Tucker BS, Coughlin ML, Figg CA, Sumerlin BS. Grafting-From Proteins Using Metal-Free PET–RAFT Polymerizations under Mild Visible-Light Irradiation. ACS Macro Letters. 2017;6(4):452-7.
