# Supplementary material for: Automated prediction of site and sequence of protein modification with ATRP initiators
Source: PLoS One. 2022 Sep 19;17(9):e0274606. doi: 10.1371/journal.pone.0274606 (PMC9484671; doi:10.1371/journal.pone.0274606)
Supplement: S3 Table — (DOCX) [file pone.0274606.s005.docx]

S3 Table PRELYM results for amine interactions on the surface of lysozyme using a probe radius equivalent to the hydrodynamic radius of oligomeric RAFT CTA (8.8 Å; see S2 File). Shaded in grey are experimental data for lysozyme from site modification studies with oligomeric RAFT CTA.[1]

| **Chain** | **Residue** | **-NH2 Group** | **ESA (Å^2^)** | **pKa** | **Secondary Structure** | **H-Donor** | **Area of Lower Charge** | **Reactivity** | |
| --- | --- | --- | --- | --- | --- | --- | --- | --- | --- |
|  |  |  |  |  |  |  |  | **Predicted** | **Experimental** |
| A | K1 | α | 205.02 | 7.43 | Coil | No | Yes | fast-reacting | modified |
|  | K1 | ε | 205.02 | 11.40 | Coil | No | Yes | fast-reacting | *not determined* |
|  | K13 | ε | 64.43 | 11.54 | Helix | Yes | Yes | slow-reacting | *not determined* |
|  | K33 | ε | 112.11 | 10.14 | Helix | Yes | Yes | slow-reacting | modified |
|  | K96 | ε | 34.66 | 10.09 | Helix | Yes | Yes | non-reacting | *not determined* |
|  | K97 | ε | 195.73 | 10.45 | Helix | No | Yes | slow-reacting | modified |
|  | K116 | ε | 218.96 | 10.06 | Coil | Yes | Yes | fast-reacting | *not determined* |

**REFERENCES**

1. Lucius M, Falatach R, McGlone C, Makaroff K, Danielson A, Williams C, et al. Investigating the Impact of Polymer Functional Groups on the Stability and Activity of Lysozyme-Polymer Conjugates. Biomacromolecules. 2016;17(3):1123-34.
