# Supplementary material for: Automated prediction of site and sequence of protein modification with ATRP initiators
Source: PLoS One. 2022 Sep 19;17(9):e0274606. doi: 10.1371/journal.pone.0274606 (PMC9484671; doi:10.1371/journal.pone.0274606)
Supplement: S4 Table — (DOCX) [file pone.0274606.s006.docx]

S4 Table PRELYM results for amine interactions on the surface of lysozyme using a probe radius approximate to the hydrodynamic radius of PEG 5 kDa[1] (17 Å). Shaded in grey are experimental data for lysozyme from site modification studies with *N*-hydroxysuccinimide PEG reagent 5 kDa.[2]

| **Chain** | **Residue** | **-NH2 Group** | **ESA (Å^2^)** | **pKa** | **Secondary Structure** | **H-Donor** | **Area of Lower Charge** | **Reactivity** | |
| --- | --- | --- | --- | --- | --- | --- | --- | --- | --- |
|  |  |  |  |  |  |  |  | **Predicted** | **Experimental** |
| A | K1 | α | 294.85 | 7.43 | Coil | No | Yes | fast-reacting | modified |
|  | K1 | ε | 294.85 | 11.40 | Coil | No | Yes | fast-reacting | *modified** |
|  | K13 | ε | 64.87 | 11.54 | Helix | Yes | Yes | slow-reacting | *modified* |
|  | K33 | ε | 177.79 | 10.14 | Helix | Yes | Yes | slow-reacting | modified |
|  | K96 | ε | 26.33 | 10.09 | Helix | Yes | Yes | non-reacting | *not determined* |
|  | K97 | ε | 285.34 | 10.45 | Helix | No | Yes | slow-reacting | modified |
|  | K116 | ε | 361.61 | 10.06 | Coil | Yes | Yes | fast-reacting | *modified* |

* Only one amine site at K1 is modified.

**REFERENCES**

1. Dong X, Al-Jumaily A, Escobar I. Investigation of the Use of a Bio-Derived Solvent for Non-Solvent-Induced Phase Separation (NIPS) Fabrication of Polysulfone Membranes. Membranes. 2018;8(2).

2. Maiser B, Dismer F, Hubbuch J. Optimization of random PEGylation reactions by means of high throughput screening. Biotechnol Bioeng. 2014;111(1):104-14.
