# Supplementary material for: Automated prediction of site and sequence of protein modification with ATRP initiators
Source: PLoS One. 2022 Sep 19;17(9):e0274606. doi: 10.1371/journal.pone.0274606 (PMC9484671; doi:10.1371/journal.pone.0274606)
Supplement: S5 Table — (DOCX) [file pone.0274606.s007.docx]

S5 Table PRELYM results for amine-ATRP initiator interactions on the surface of homo-dimer chymotrypsin. Shaded in grey are the experimental reactivity data for chymotrypsin from site modification studies with *N*-hydroxysuccinimide ATRP initiator.[1]

| **Chain** | **Residue** | **-NH2 Group** | **ESA (Å^2^)** | **pKa** | **Secondary Structure** | **H-Donor** | **Area of Lower Charge** | **Reactivity** | |
| --- | --- | --- | --- | --- | --- | --- | --- | --- | --- |
|  |  |  |  |  |  |  |  | **Predicted** | **Experimental** |
| A | C1 | α | 126.0 | 7.66 |  | No |  | fast-reacting | fast-reacting |
| B | I16 | α | 0 |  |  | No |  | non-reacting | *not determined* |
|  | K36 | ε | 131.57 | 10.46 | Coil | No | No | slow-reacting* | fast-reacting |
|  | K79 | ε | 275.10 | 10.45 | Coil | No | Yes | fast-reacting | slow-reacting |
|  | K82 | ε | 120.04 | 10.36 | Strand | No | No | slow-reacting | non-reacting |
|  | K84 | ε | 184.79 | 10.41 | Strand | No | Yes | slow-reacting | *not determined* |
|  | K87 | ε | 181.62 | 10.24 | Strand | Yes | Yes | fast-reacting | *not determined* |
|  | K90 | ε | 86.98 | 10.12 | Strand | No | Yes | fast-reacting | slow-reacting |
|  | K93 | ε | 217.84 | 10.41 | Coil | Yes | Yes | fast-reacting | *not determined* |
|  | K107 | ε | 53.90 | 10.79 | Strand | Yes | No | slow-reacting | non-reacting |
| C | A149 | α | 5.50 | 7.54 |  | No |  | non-reacting | *not determined* |
|  | K169 | ε | 67.26 | 10.37 | Helix | Yes | Yes | slow-reacting | slow-reacting |
|  | K170 | ε | 286.60 | 10.49 | Helix | Yes | Yes | non-reacting | non-reacting |
|  | K175 | ε | 32.42 | 9.89 | Helix | Yes | Yes | non-reacting | *not determined* |
|  | K177 | ε | 57.55 | 10.13 | Coil | Yes | Yes | slow-reacting | slow-reacting |
|  | K202 | ε | 104.07 | 10.22 | Strand | No | Yes | fast-reacting | fast-reacting |
|  | K203 | ε | 58.88 | 10.74 | Strand | Yes | Yes | non-reacting | non-reacting |

**REFERENCES**

1. Carmali S, Murata H, Amemiya E, Matyjaszewski K, Russell AJ. Tertiary Structure-Based Prediction of How ATRP Initiators React with Proteins. ACS Biomaterials Science & Engineering. 2017;3(9):2086-97.
