# Supplementary material for: Automated prediction of site and sequence of protein modification with ATRP initiators
Source: PLoS One. 2022 Sep 19;17(9):e0274606. doi: 10.1371/journal.pone.0274606 (PMC9484671; doi:10.1371/journal.pone.0274606)
Supplement: S7 Table — (DOCX) [file pone.0274606.s009.docx]

S7 Table PRELYM results for amine interactions on the surface of monomer chymotrypsin using a probe radius approximate to the hydrodynamic radius of PEG 5 kDa [1] (17 Å).

| **Chain** | **Residue** | **-NH2 Group** | **ESA (Å^2^)** | **pKa** | **Secondary Structure** | **H-Donor** | **Area of Lower Charge** |  |
| --- | --- | --- | --- | --- | --- | --- | --- | --- |
|  |  |  |  |  |  |  |  | **Predicted** |
| A | C1 | α | 169.61 | 7.66 |  | No |  | fast-reacting |
| B | I16 | α | 0 |  |  | No |  | non-reacting |
|  | K36 | ε | 673.34 | 10.45 | Coil | No | No | slow-reacting* |
|  | K79 | ε | 754.37 | 10.45 | Coil | No | Yes | fast-reacting |
|  | K82 | ε | 46.57 | 10.36 | Strand | No | No | non-reacting |
|  | K84 | ε | 439.01 | 10.41 | Strand | No | Yes | slow-reacting |
|  | K87 | ε | 421.02 | 10.24 | Strand | Yes | Yes | fast-reacting |
|  | K90 | ε | 131.41 | 10.33 | Strand | No | Yes | slow-reacting |
|  | K93 | ε | 538.57 | 10.41 | Coil | Yes | Yes | fast-reacting |
|  | K107 | ε | 39.50 | 10.79 | Strand | Yes | No | non-reacting |
| C | A149 | α | 627.86 | 7.89 |  | No |  | fast-reacting |
|  | K169 | ε | 17.52 | 10.37 | Helix | Yes | Yes | non-reacting |
|  | K170 | ε | 1046.5 | 10.49 | Helix | Yes | Yes | slow-reacting |
|  | K175 | ε | 179.85 | 10.25 | Helix | Yes | Yes | slow-reacting |
|  | K177 | ε | 9.38 | 10.10 | Coil | Yes | Yes | non-reacting |
|  | K202 | ε | 86.31 | 10.22 | Strand | No | Yes | fast-reacting |
|  | K203 | ε | 4.52 | 10.74 | Strand | Yes | Yes | non-reacting |

**REFERENCES**

1. Dong X, Al-Jumaily A, Escobar I. Investigation of the Use of a Bio-Derived Solvent for Non-Solvent-Induced Phase Separation (NIPS) Fabrication of Polysulfone Membranes. Membranes. 2018;8(2).
