# Supplementary material for: Automated prediction of site and sequence of protein modification with ATRP initiators
Source: PLoS One. 2022 Sep 19;17(9):e0274606. doi: 10.1371/journal.pone.0274606 (PMC9484671; doi:10.1371/journal.pone.0274606)
Supplement: S8 Table — (DOCX) [file pone.0274606.s010.docx]

S8 Table PRELYM results for amine-ATRP initiator interactions on the surface of monomer glucose oxidase.

| **Chain** | **Residue** | **-NH2 Group** | **ESA (Å^2^)** | **pKa** | **Secondary Structure** | **H-Donor** | **Area of Lower Charge** | **Predicted**  **Reactivity** |
| --- | --- | --- | --- | --- | --- | --- | --- | --- |
| A | S1 | α | 320.65 | 7.90 |  | No |  | fast-reacting |
|  | K13 | ε | 210.81 | 10.35 | Helix | Yes | No | slow-reacting |
|  | K116 | ε | 97.34 | 10.40 | Helix | Yes | No | slow-reacting |
|  | K152 | ε | 50.32 | 11.31 | Helix | Yes | No | slow-reacting |
|  | K187 | ε | 97.76 | 10.19 | Helix | No | No | slow-reacting |
|  | K201 | ε | 56.25 | 9.79 | Coil | Yes | No | slow-reacting |
|  | K202 | ε | 98.19 | 10.42 | Coil | No | No | non-reacting |
|  | K252 | ε | 38.57 | 10.22 | Strand | No | No | non-reacting |
|  | K273 | ε | 196.68 | 10.54 | Coil | No | No | slow-reacting |
|  | K282 | ε | 142.90 | 10.58 | Strand | Yes | No | slow-reacting |
|  | K306 | ε | 176.18 | 10.44 | Helix | Yes | No | slow-reacting |
|  | K364 | ε | 56.75 | 10.08 | Helix | No | No | slow-reacting |
|  | K372 | ε | 68.69 | 10.48 | Helix | Yes | No | slow-reacting |
|  | K441 | ε | 137.09 | 10.30 | Coil | Yes | No | fast-reacting |
|  | K526 | ε | 90.31 | 10.13 | Helix | No | No | slow-reacting |
|  | K570 | ε | 0 | 8.61 | Helix | Yes | No | non-reacting |
