# Supplementary material for: Automated prediction of site and sequence of protein modification with ATRP initiators
Source: PLoS One. 2022 Sep 19;17(9):e0274606. doi: 10.1371/journal.pone.0274606 (PMC9484671; doi:10.1371/journal.pone.0274606)
Supplement: S9 Table — (DOCX) [file pone.0274606.s011.docx]

S9 Table PRELYM results for amine-ATRP initiator interactions on the surface of dimer avidin. Shaded in grey are the experimental data for avidin from site modification studies with *N*-hydroxysuccinimide ATRP initiator.[1]

| **Chain** | **Residue** | **-NH2 Group** | **ESA (Å^2^)** | **pKa** | **Secondary Structure** | **H-Donor** | **Area of Lower Charge** | **Predicted** | **Experimental Modification** |
| --- | --- | --- | --- | --- | --- | --- | --- | --- | --- |
|  |  |  |  |  |  |  |  | **Reactivity** |  |
| A | A1 | α | 84.86 | 6.65 |  | No |  | fast-reacting | *not determined* |
|  | K3 | ε | 229.99 | 10.41 | Coil | No | Yes | fast-reacting | *not determined* |
|  | K9 | ε | 41.99 | 10.28 | Strand | No | Yes | non-reacting | *not determined* |
|  | K45 | ε | 143.07 | 10.39 | Coil | No | Yes | fast-reacting | modified |
|  | K58 | ε | 210.76 | 10.20 | Coil | No | Yes | fast-reacting | *not determined* |
|  | K71 | ε | 137.21 | 10.12 | Coil | Yes | Yes | fast-reacting | modified |
|  | K90 | ε | 123.78 | 10.47 | Coil | No | Yes | fast-reacting | *not determined* |
|  | K94 | ε | 18.43 | 10.02 | Strand | Yes | Yes | non-reacting | *not determined* |
|  | K111 | ε | 120.41 | 10.39 | Helix | No | Yes | slow-reacting | modified |
|  | K127 | ε | 286.97 | 10.46 | Coil | No |  | slow-reacting | *not determined* |
| B | A1 | α | 114.08 | 7.68 |  | No |  | fast-reacting | *not determined* |
|  | K3 | ε | 136.65 | 10.45 | Coil | No | Yes | fast-reacting | *not determined* |
|  | K9 | ε | 172.78 | 10.35 | Strand | No | Yes | slow-reacting | *not determined* |
|  | K45 | ε | 151.79 | 10.55 | Coil | No | Yes | fast-reacting | modified |
|  | K58 | ε | 152.60 | 10.11 | Coil | No | Yes | fast-reacting | *not determined* |
|  | K71 | ε | 187.98 | 10.26 | Coil | Yes | Yes | fast-reacting | modified |
|  | K90 | ε | 70.92 | 10.36 | Coil | No | Yes | slow-reacting | *not determined* |
|  | K94 | ε | 30.96 | 10.23 | Strand | Yes | Yes | non-reacting | *not determined* |
|  | K111 | ε | 118.67 | 10.27 | Helix | No | Yes | slow-reacting | modified |
|  | K127 | ε | 329.13 | 10.47 | Coil | No |  | slow-reacting | *not determined* |

**REFERENCES**

1. Kaupbayeva B, Murata H, Lucas A, Matyjaszewski K, Minden JS, Russell AJ. Molecular Sieving on the Surface of a Nano-Armored Protein. Biomacromolecules. 2019;20(3):1235-45.
