# Supplementary material for: Automated prediction of site and sequence of protein modification with ATRP initiators
Source: PLoS One. 2022 Sep 19;17(9):e0274606. doi: 10.1371/journal.pone.0274606 (PMC9484671; doi:10.1371/journal.pone.0274606)
Supplement: S10 Table — (DOCX) [file pone.0274606.s012.docx]

S10 Table PRELYM results for amine-ATRP initiator interactions on the surface of interferon-α 2a. Shaded in grey are experimental data obtained from site modification studies with PEGylated interferon-α 2a using an amine-reactive branched 40 kDa PEG.[1]

| **Chain** | **Residue** | **-NH2 Group** | **ESA (Å^2^)** | **pKa** | **Secondary Structure** | **H-Donor** | **Area of Lower Charge** | **Predicted**  **Reactivity** | **PEGylation Sites in INF-α 2a** |
| --- | --- | --- | --- | --- | --- | --- | --- | --- | --- |
| A | C1 | α | 36.01 | 7.68 |  | No |  | non-reacting | not modified |
|  | K23 | ε | 103.84 | 10.67 | Coil | Yes | No | fast-reacting | not modified |
|  | K31 | ε | 287.07 | 10.44 | Coil | Yes | Yes | fast-reacting | modified |
|  | K49 | ε | 187.51 | 11.16 | Coil | Yes | No | fast-reacting | modified |
|  | K70 | ε | 276.63 | 10.62 | Helix | No | Yes | slow-reacting | modified |
|  | K83 | ε | 94.70 | 10.29 | Helix | Yes | Yes | slow-reacting | modified |
|  | K112 | ε | 7.32 | 10.09 | Helix | No | No | non-reacting | modified |
|  | K121 | ε | 134.93 | 10.36 | Helix | No | No | slow-reacting | modified |
|  | K131 | ε | 143.79 | 11.56 | Helix | No | Yes | slow-reacting | modified |
|  | K133 | ε | 29.34 | 11.09 | Coil | Yes | Yes | non-reacting | not modified |
|  | K134 | ε | 304.32 | 10.42 | Coil | Yes | Yes | fast-reacting | modified |
|  | K164 | ε | 172.67 | 10.37 | Coil | No | Yes | fast-reacting | modified |

**REFERENCES**

1. Foser S, Schacher A, Weyer KA, Brugger D, Dietel E, Marti S, et al. Isolation, structural characterization, and antiviral activity of positional isomers of monopegylated interferon α-2a (PEGASYS). Protein Expression and Purification. 2003;30(1):78-87.
