# Supplementary material for: Automated prediction of site and sequence of protein modification with ATRP initiators
Source: PLoS One. 2022 Sep 19;17(9):e0274606. doi: 10.1371/journal.pone.0274606 (PMC9484671; doi:10.1371/journal.pone.0274606)
Supplement: S12 Table — (DOCX) [file pone.0274606.s014.docx]

S12 Table PRELYM results for amine-ATRP initiator interactions on the surface of asparaginase II.

| **Chain** | **Residue** | **-NH2 Group** | **ESA (Å^2^)** | **pKa** | **Secondary Structure** | **H-Donor** | **Area of Lower Charge** | **Predicted**  **Reactivity** |
| --- | --- | --- | --- | --- | --- | --- | --- | --- |
| A | L1 | α | 207.88 | 7.85 |  | No |  | fast-reacting |
|  | K22 | ε | 122.08 | 10.47 | Coil | No | No | slow-reacting |
|  | K29 | ε | 137.77 | 10.34 | Coil | Yes | No | fast-reacting |
|  | K43 | ε | 128.70 | 10.28 | Helix | No | No | slow-reacting |
|  | K49 | ε | 92.84 | 10.38 | Strand | Yes | No | slow-reacting |
|  | K71 | ε | 22.037 | 9.99 | Helix | Yes | No | non-reacting |
|  | K72 | ε | 30.52 | 12.22 | Helix | Yes | No | non-reacting |
|  | K79 | ε | 155.17 | 10.97 | Helix | Yes | No | slow-reacting |
|  | K104 | ε | 46.36 | 8.88 | Coil | Yes | No | non-reacting |
|  | K107 | ε | 56.99 | 10.26 | Coil | Yes | No | slow-reacting |
|  | K139 | ε | 268.35 | 10.47 | Helix | No | No | slow-reacting |
|  | K162 | ε | 0 | 10.11 | Strand | Yes | No | non-reacting |
|  | K172 | ε | 1.26 | 10.36 | Strand | Yes | No | non-reacting |
|  | K186 | ε | 91.10 | 10.74 | Strand | Yes | No | slow-reacting |
|  | K196 | ε | 124.97 | 11.42 | Coil | Yes | No | fast-reacting |
|  | K207 | ε | 315.16 | 10.50 | Coil | No | No | slow-reacting |
|  | K213 | ε | 71.43 | 10.63 | Coil | Yes | No | slow-reacting |
|  | K229 | ε | 93.55 | 10.81 | Helix | Yes | No | slow-reacting |
|  | K251 | ε | 133.65 | 10.38 | Helix | No | No | slow-reacting |
|  | K262 | ε | 194.76 | 10.26 | Helix | No | No | slow-reacting |
|  | K288 | ε | 197.36 | 11.17 | Helix | No | No | slow-reacting |
|  | K301 | ε | 0 | 8.28 | Helix | Yes | No | non-reacting |
|  | K314 | ε | 166.0 | 10.51 | Coil | No | No | slow-reacting |
