# Supplementary material for: Automated prediction of site and sequence of protein modification with ATRP initiators
Source: PLoS One. 2022 Sep 19;17(9):e0274606. doi: 10.1371/journal.pone.0274606 (PMC9484671; doi:10.1371/journal.pone.0274606)
Supplement: S13 Table — (DOCX) [file pone.0274606.s015.docx]

S13 Table PRELYM results for amine interactions on the surface of asparaginase II using a probe radius approximate to the hydrodynamic radius of PEG 5 kDa [1] (17 Å).

| **Chain** | **Residue** | **-NH2 Group** | **ESA (Å^2^)** | **pKa** | **Secondary Structure** | **H-Donor** | **Area of Lower Charge** | **Predicted**  **Reactivity** |
| --- | --- | --- | --- | --- | --- | --- | --- | --- |
| A | L1 | α | 419.19 | 7.85 |  | No |  | fast-reacting |
|  | K22 | ε | 75.66 | 10.47 | Coil | No | No | slow-reacting |
|  | K29 | ε | 132.54 | 10.34 | Coil | Yes | No | fast-reacting |
|  | K43 | ε | 233.36 | 10.28 | Helix | No | No | slow-reacting |
|  | K49 | ε | 67.31 | 10.38 | Strand | Yes | No | slow-reacting |
|  | K71 | ε | 0 | 9.99 | Helix | Yes | No | non-reacting |
|  | K72 | ε | 1.88 | 12.22 | Helix | Yes | No | non-reacting |
|  | K79 | ε | 434.17 | 10.97 | Helix | Yes | No | slow-reacting |
|  | K104 | ε | 0 | 8.88 | Coil | Yes | No | non-reacting |
|  | K107 | ε | 5.57 | 10.26 | Coil | Yes | No | non-reacting |
|  | K139 | ε | 509.46 | 10.47 | Helix | No | No | slow-reacting |
|  | K162 | ε | 0 | 10.11 | Strand | Yes | No | non-reacting |
|  | K172 | ε | 0 | 10.36 | Strand | Yes | No | non-reacting |
|  | K186 | ε | 0 | 10.74 | Strand | Yes | No | non-reacting |
|  | K196 | ε | 136.21 | 11.42 | Coil | Yes | No | fast-reacting |
|  | K207 | ε | 972.58 | 10.50 | Coil | No | No | slow-reacting |
|  | K213 | ε | 0 | 10.63 | Coil | Yes | **No** | non-reacting |
|  | K229 | ε | 195.24 | 10.81 | Helix | Yes | No | slow-reacting |
|  | K251 | ε | 167.62 | 10.38 | Helix | No | No | slow-reacting |
|  | K262 | ε | 351.93 | 10.26 | Helix | No | No | slow-reacting |
|  | K288 | ε | 419.62 | 11.17 | Helix | No | No | slow-reacting |
|  | K301 | ε | 0 | 8.28 | Helix | Yes | No | non-reacting |
|  | K314 | ε | 349.18 | 10.51 | Coil | No | No | slow-reacting |

**REFERENCES**

1. Linegar KL, Adeniran AE, Kostko AF, Anisimov MA. Hydrodynamic radius of polyethylene glycol in solution obtained by dynamic light scattering. Colloid Journal. 2010;72(2):279-81.
