# Supplementary material for: Automated prediction of site and sequence of protein modification with ATRP initiators
Source: PLoS One. 2022 Sep 19;17(9):e0274606. doi: 10.1371/journal.pone.0274606 (PMC9484671; doi:10.1371/journal.pone.0274606)
Supplement: S14 Table — (DOCX) [file pone.0274606.s016.docx]

S14 Table PRELYM results for amine-ATRP initiator interactions on the surface of tetrameric phenylalanine ammonia lyase (rAV-PAL). Shaded in grey are experimental data obtained for rAV-PAL from site modification studies using a 20 kDa *N*-hydroxysuccinimide PEG.[1] Percentage of modification was determined by mass spectrometry after tryptic digestion.

| **Chain** | **Residue** | **-NH2 Group** | **ESA (Å^2^)** | **pKa** | **Secondary Structure** | **H-Donor** | **Area of Lower Charge** | **Predicted**  **Reactivity** | **PEGylation Sites**  **(% of modification)** |
| --- | --- | --- | --- | --- | --- | --- | --- | --- | --- |
|  | M1 | α | 468.51 | 7.93 |  | No |  | fast-reacting | *not determined* |
|  | K2 | ε | 270.99 | 10.41 | Coil | No |  | non-reacting | modified (100%) |
|  | K10 | ε | 282.00 | 10.46 | Coil | No |  | slow-reacting | modified (100%) |
|  | K32 | ε | 111.01 | 9.93 | Coil | No | No | slow-reacting | modified (40%) |
|  | K109 | ε | 0 | 10.18 | Coil | Yes | No | non-reacting | *not determined* |
|  | K115 | ε | 46.99 | 7.95 | Strand | No | No | non-reacting | modified (20%) |
|  | K145 | ε | 65.75 | 10.52 | Helix | Yes | No | slow-reacting | modified (50%) |
|  | K189 | ε | 38.13 | 10.44 | Strand | Yes | No | non-reacting | *not determined* |
|  | K195 | ε | 254.07 | 10.41 | Strand | Yes | No | slow-reacting | modified (100%) |
|  | K216 | ε | 0.109 | 9.18 | Coil | Yes | No | non-reacting | *not determined* |
|  | K272 | ε | 0 | 9.55 | Coil | Yes | No | non-reacting | *not determined* |
|  | K301 | ε | 171.12 | 10.60 | Coil | Yes | No | fast-reacting | modified (40%) |
|  | K335 | ε | 107.23 | 10.10 | Helix | Yes | No | slow-reacting | modified (20%) |
|  | K384 | ε | 0 | 5.89 | Helix | Yes | No | non-reacting | *not determined* |
|  | K413 | ε | 170.57 | 10.37 | Coil | No | No | slow-reacting | modified (90%) |
|  | K419 | ε | 0 | 10.68 | Helix | Yes | No | non-reacting | modified (20%) |
|  | K493 | ε | 147.60 | 9.98 | Helix | Yes | No | slow-reacting | *modified (100%) |
|  | K494 | ε | 124.47 | 10.34 | Helix | Yes | No | non-reacting | *modified (100%) |
|  | K522 | ε | 230.93 | 10.42 | Coil | No | No | slow-reacting | modified (100%) |

**REFERENCES**

1. Bell SM, Wendt DJ, Zhang Y, Taylor TW, Long S, Tsuruda L, et al. Formulation and PEGylation optimization of the therapeutic PEGylated phenylalanine ammonia lyase for the treatment of phenylketonuria. PLOS ONE. 2017;12(3):e0173269.
